# Description of AtCAX4 in Response to Abiotic Stress in Arabidopsis

**DOI:** 10.3390/ijms22020856

**Published:** 2021-01-16

**Authors:** Yuanyuan Bu, Weichao Fu, Jiangpo Chen, Tetsuo Takano, Shenkui Liu

**Affiliations:** 1 Key Laboratory of Saline-Alkali Vegetation Ecology Restoration (SAVER), Ministry of Education, Alkali Soil Natural Environmental Science Center (ASNESC), Northeast Forestry University, Harbin 150040, China; yuanyuanbu@nefu.edu.cn (Y.B.); wcfu2020@163.com (W.F.); jpchen20162020@163.com (J.C.); 2 Asian Natural Environmental Science Center (ASNESC), The University of Tokyo, Nishitokyo, Tokyo 188-0002, Japan; takano@anesc.u-tokyo.ac.jp; 3 State Key Laboratory of Subtropical Silviculture, Zhejiang A & F University, Lin’an, Hangzhou 311300, China

**Keywords:** Ca^2+^/H^+^ antiporters (CAXs), interaction, abiotic stress

## Abstract

High-capacity tonoplast cation/H^+^ antiport in plants is partially mediated by a family of CAX transporters. Previous studies have reported that CAX activity is affected by an N-terminal autoinhibitory region. CAXs may be present as heterodimers in plant cells, and this phenomenon necessitates further study. In this study, we demonstrate that there is an interaction between CAX4 and CAX1 as determined by the use of a yeast two-hybrid system and a bimolecular fluorescence complementation assay. More specifically, the N-terminal of CAX4 interacts with CAX1. We further observed the over-expression and either a single or double mutant of *CAX1* and *CAX4* in response to abiotic stress in *Arabidopsis*. These results suggest that CAX1 and CAX4 can interact to form a heterodimer, and the N-terminal regions of CAX4 play important roles in vivo; this may provide a foundation for a deep study of CAX4 function in the future.

## 1. Introduction

Calcium (Ca^2+^) ions function as a ubiquitous signal within cells and have been reported to play an important role in many processes in plant cells and they appear to be involved in several aspects of plant growth and development, as well as the adaptive response of plants to abiotic and biotic stimuli [1,2,3,4]. Cytosolic Ca^2+^ increases in response to environmental stress and the Ca^2+^ efflux system plays a critical role in restoring basal cytosolic Ca^2+^ levels and terminating stress-induced cytosolic Ca^2+^ signatures [5,6]. Tonoplast-localized Ca^2+^/H^+^ antiporters (CAXs) play a critical role in sequestering Ca^2+^ into the vacuole and thus contribute to Ca^2+^ homeostasis in plant cells [7,8]. CAXs are membrane proteins that export Ca^2+^ and other cations by utilizing an H^+^ gradient established by H^+^-ATPase or H^+^-pyrophosphatase [9]. CAXs are members of a multigene family and have been identified in many species. Research on CAXs over the past several years have focused on their physiological role, biochemistry, and molecular biology. Studies on CAXs include (1) their ability to transport trace metal ions, as well as Ca^2+^ (CAX transporters have been reported to function as cation selectivity filters, with some CAX isoforms having a broad range of cation specificity, where the specificity is dependent on the amino acid sequence of the different CAXs) [7,10,11,12,13,14,15,16,17]; (2) the expression pattern of CAX genes in response to diverse types of stresses; (3) the participation of CAXs in several aspects of plant growth and development under stress conditions [18]; as well as several other aspects. In addition to our current understanding of the biochemistry and function of CAXs, further studies on the N-terminal autoinhibition of CAXs and CAX protein-forming complexes are still needed.

A putative N-terminal autoinhibitory region in CAXs has been identified in Arabidopsis, rice, and mung bean [15,19,20,21]. Deletions of the N-terminal region increase CAX activity in both yeast and plants [7,22]. CAX1 acts as a weak vacuolar Ca^2+^⁄H^+^ antiporter in yeast; however, transport activity is severely reduced relative to sCAX1 (N-terminally truncated form of CAX1) [12,23]. This autoinhibition is caused by the physical interaction of the N-terminus with a neighboring N terminal region (residues 56–62) [7]. Our previous studies have shown that expression of the N-terminally truncated form of *PutCAX1*, a CAX from *Puccinellia tenuiflora*, can complement active Ca^2+^ transporters and confer Ba^2+^ tolerance to yeast [13]. These findings suggest that the N-terminus of CAXs acts as an auto-inhibitory domain for cation/H^+^ transport activity in yeast and plants.

The *Arabidopsis thaliana* genome appears to contain six CAX genes, designated *AtCAX1–AtCAX6* [7]. Members of the Arabidopsis CAX gene family, such as CAX1, CAX2, and CAX3, have been characterized at both the molecular and whole-plant level [12,19,20,23,24,25,26]. *CAX1* is most closely related to *CAX3* at the amino acid level and, together with *CAX4,* belong to type I-A CAX family genes [7,11,27]. *CAX4* has been partially characterized biochemically through heterologous expression in yeast and tobacco to determine its cation transport properties [26,28]. Recent studies have focused on the function of CAX transporters that are less highly expressed. For example, *AtCAX4* is expressed in the root apex and in lateral root primordia, and the level of *CAX4* mRNA was reported to increase in response to Mn^2+^, Na^+^, and Ni^+^ treatments [17,28]. This pattern of root expression appears to be unique among the different Arabidopsis CAXs. Transgenic plants expressing increased levels of *CAX4* display symptoms consistent with increased vacuolar sequestration of Ca^2+^ and Cd^2+^. High levels of expression of *CAX4* in an Arabidopsis *cax1* mutant line with weak vacuolar Ca^2+^/H^+^ antiport activity results in a 29% increase in Ca^2+^/H^+^ antiport activity. A *cax4* loss-of-function mutant and *CAX4* RNA interference (*CAX4* RNAi) lines display altered root growth and development in response to Cd^2+^, Mn^2+^, and auxin [17]. These findings indicate that CAX4 is a cation⁄H^+^ antiporter that plays an important functional role in root growth under abiotic stress conditions [17].

In-depth genetic studies of CAX family members have provided new information that suggests that CAXs play overlapping roles in many cell functions. For example, plants that have lost a single CAX transport function are almost indistinguishable from wild-type plants under normal growth conditions [7]. However, when some double *CAX* deletions are constructed, the resulting plants display severe growth defects, implying the importance and compensatory function of CAXs [18,25]. In addition, the co-expression of full-length *CAX1* and *CAX3* in yeast results in the production of functional transporters with different biochemical properties than *CAX1* alone [29]. Native CAX exchangers, represented by CAX1 and CAX3 polypeptides, may alter the function of a protomembrane in comparison to dimer transporters composed only of the CAX1 coding sequence. In some cases, the properties of the oligomer can affect their function [30]. Previous work with ammonium transporters in Arabidopsis suggests that allosteric interactions between isoforms may be essential for activity [31]. In some cases, coupling between transporters may represent a mechanism for increasing the dynamic range of transporter regulation and function. Hocking et al. [32] conducted a combined transshipment and biochemical analysis of CAX heterologous dimers using laser capture microdissection combined with single-cell RNA analysis. Bimolecular fluorescence complementation (BiFC) analysis of CAX1:CAX3 demonstrated that these CAX isoforms are capable of forming dimers [32]. Thus, CAXs may function as heterodimers in plants.

In the present study, we have analyzed the interaction between the Arabidopsis Ca^2+^/H^+^ exchangers CAX4 and CAX1. Using a yeast two-hybrid system and BiFC assay, we demonstrate that there is an interaction between full-length CAX4 and CAX1. More specifically, the N-terminus of CAX4 interacts with CAX1. Then, we analyzed the responses of *CAX1* and *CAX4* to abiotic stress in plants using over-expression or the use of single and double mutants of *CAX1* and *CAX4*. Our results suggest that CAX1 and CAX4 may form a heterodimer in plants in response to abiotic stress.

## 2. Results

### 2.1. AtCAX1 Interacts with the N-Terminus of AtCAX4

**Figure 1:**
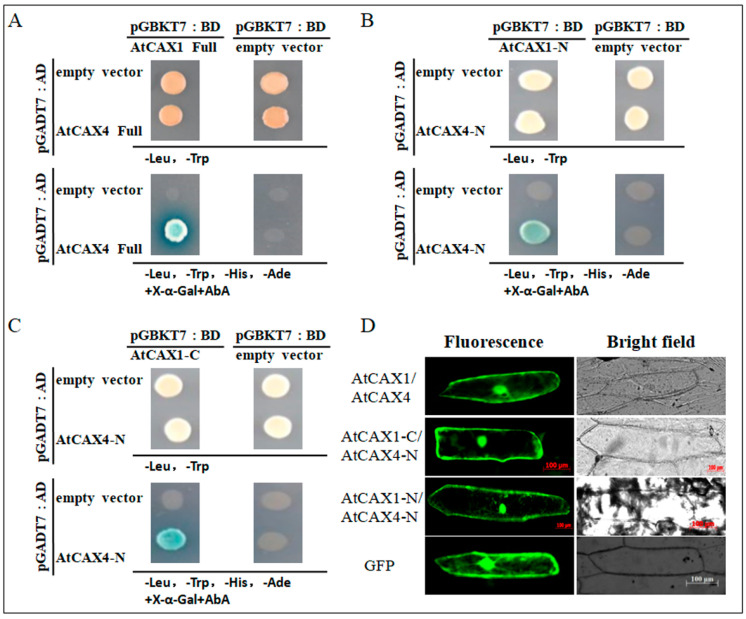
AtCAX1 interacts with AtCAX4. (**A**–**C**) Yeast two-hybrid assay. The combinations of plasmids: full length of pGBKT7-AtCAX1 and pGADT7-AtCAX4 (**A**), C-terminus of pGBKT7-AtCAX1 and N-terminus sequence of pGADT7-AtCAX4 (**B**), N-terminus of pGBKT7-AtCAX1 and N-terminus sequences of pGADT7-AtCAX4 (**C**) were co-transformed into yeast Y2HGold cells. Transformed yeast cells were grown on SD-Leu-Trp and SD-Leu-Trp-His-Ade+X-a-gal+AbA medium for 2–3 days at 30 °C. (**D**) Bimolecular fluorescence complementation (BiFC) in onion epidermal cells. The combinations of plasmids (full length of AtCAX1-nGFP and AtCAX4-cGFP, C-terminus of AtCAX1 and N-terminus of AtCAX4, N-terminus of AtCAX1 and N-terminus of AtCAX4) are shown on the left. GFP was used a control. Scale bar = 100 μm.

Interactions between proteins or between proteins and nucleic acids are often required for functional activity in many biolgical processes. AtCAX4, along with AtCAX1, belong to type I-A CAX family proteins [7] and play an important role in Ca^2+^ homeostasis [13]. AtCAX4 and AtCAX1 were co-transformed into Y2HGold strains as part of a yeast two-hybrid system to explore their potential interactions and to better understand the function of AtCAX4 protein in Ca^2+^ ion balance in plants. Results indicated that AtCAX4 and AtCAX1 do interact with each other. Then, full-length *AtCAX1* and *AtCAX4* or N-truncated *AtCAX1* (*∆NAtCAX1* or *AtCAX1-C*), C-truncated *AtCAX1* (*∆CAtCAX1* or *AtCAX1-N*), N-truncated *AtCAX4* (*∆NAtCAX4* or *AtCAX4-C*), and C-truncated *AtCAX4* (*∆CAtCAX4* or *AtCAX4-N*) were cotransformed into Y2HGold strains to confirm the interaction. The obtained results demonstrated that full-length *AtCAX1* and *AtCAX4* produce blue colonies, thus confirming their ability to interact with each other (Figure 1A). Blue colonies were also formed when the N-terminal sequences of *AtCAX1* (*AtCAX1-N*) or C-terminal sequences of *AtCAX1* (*AtCAX1-C*), and the N-terminal sequence of *AtCAX4* (*AtCAX4-N*) were present. However, the presence of only C-terminal sequences of *AtCAX1* (*AtCAX1-C*) and the C-terminal sequence of *AtCAX4* (*AtCAX4-C*) did not produce blue colonies (Figure S1), indicating that AtCAX1 interacts with the N-terminus of AtCAX4 (Figure 1B,C).

The interaction between AtCAX1 and AtCAX4 was verified in vivo using a BiFC assay. Green fluorescence was observed when AtCAX1-nGFP and AtCAX4-cGFP, or when the N-terminal sequences of AtCAX1-nGFP (AtCAX1-N) or C-terminal sequences of AtCAX1-nGFP (AtCAX1-C) and the N-terminal sequence of AtCAX4-cGFP (AtCAX4-N) were expressed together in onion cells (Figure 1D). These results demonstrate that AtCAX1 can interact with the N-terminal sequence of AtCAX4 in an endomembrane system in vivo. The observed localization pattern was also consistent with findings reported in a previous study [28].

### 2.2. Expression of AtCAX1 and AtCAX4 in Response to Salt and Ion Stress

**Figure 2:**
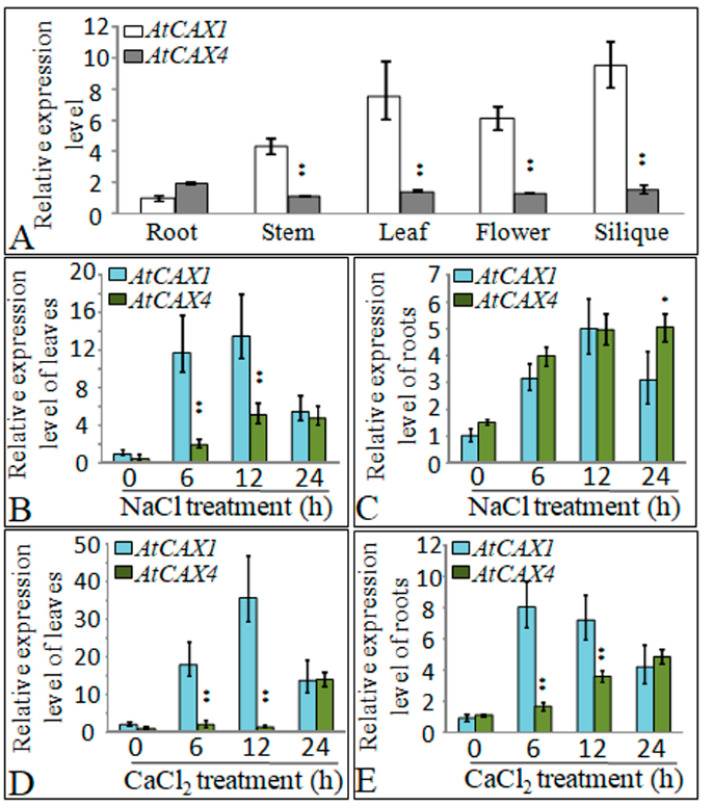
Expression of *AtCAX1* and *AtCAX4* in plant tissues subjected to stress conditions. (**A**) RT-qPCR analysis of *AtCAX1* and *AtCAX4* in different organs of *A. thaliana*. (**B**–**E**) RT-qPCR analysis of the expression of *AtCAX1* and *AtCAX4* in response to various abiotic stresses (see Methods for details). *AtCAX1* and *AtCAX4* expression was normalized against Actin mRNA levels. The reported data are the means ± SE of three replicate experiments. Single and double asterisks indicate significant differences from wild type (WT) at *p* < 0.05 and *p* < 0.01, respectively.

RNA was extracted from root, stem, leaf, flower, and silique tissues of Arabidopsis plants grown for 3 weeks under favorable growth conditions to investigate the pattern of AtCAX1 and AtCAX4 expression. Reverse transcription quantitative PCR (RT-qPCR) analysis conducted on various tissues indicated that AtCAX1 and AtCAX4 are expressed in all Arabidopsis plant organs, with the expression level of AtCAX1 being higher in silique and leaf tissues, while AtCAX4 was expressed more highly in root tissues (Figure 2A). The level of expression of AtCAX1 and AtCAX4 mRNA in response to salt and ion stress was also assessed (Figure 2B–E). AtCAX1 mRNA peaked at 12 h in response to NaCl and then declined at 24 h in both leaves and roots, while AtCAX4 mRNA increased gradually in leaves and roots in response to NaCl (Figure 2B,C). AtCAX1 gene expression was also induced in response to CaCl_2_ and peaked at 12 h or 6 h after treatment in leaf and root tissues, respectively, and then declined. In contrast, AtCAX4 mRNA expression gradually increased in both leaf and root tissues, peaking at 24 h (Figure 2D,E). These results indicate that AtCAX1 and AtCAX4 are involved in plant response to salt and ion stress.

### 2.3. Loss of Function Mutants of AtCAX1 and AtCAX4 Render Arabidopsis Plants Sensitive to Salt and Ion Stress

A T-DNA insertion located in the third exon of AtCAX1 and eight introns in AtCAX4 (Figure S2A) was confirmed by PCR-based genotype analysis (Figure S2B). PCR analysis was performed on the homozygous T-DNA insertion atcax1 and atcax4 mutants. The analysis demonstrated that they completely lack AtCAX1 or AtCAX4 transcripts (Figure S2B). F2 homozygous progeny double mutants atcax1/atcax4 were also identified by PCR analysis, and further analysis also indicated that they completely lack AtCAX1 and AtCAX4 transcripts (Figure S2B). Three Arabidopsis transgenic lines overexpressing AtCAX1 or AtCAX4 were generated under the control of the CaMV35S promoter (#1–#3) and identified by northern blotting (Figure S2C). Control samples demonstrated weak AtCAX1 or AtCAX4 signals, while the transgenic plants exhibited strong signals, indicating that they had been successfully transformed with AtCAX1 or AtCAX4.

**Figure 3:**
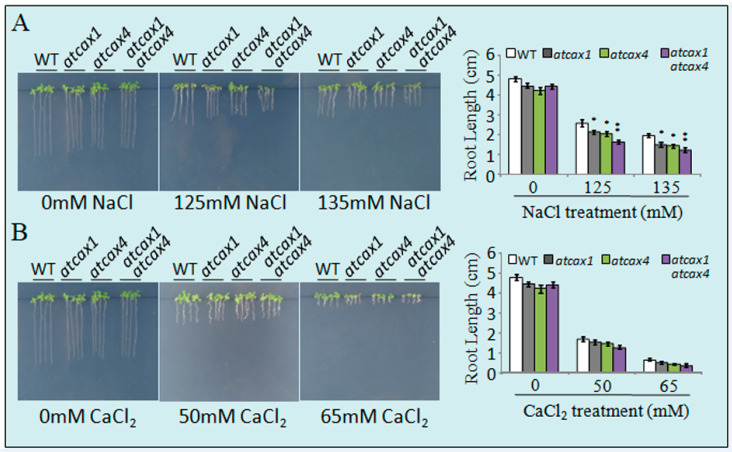
Stress sensitivity of wild-type (WT) and *atcax1*, *atcax4*, and *atcax1/atcax4* plants. Seeds were germinated on half-strength Murashige and Skoog (MS) medium containing either 125 or 135 mM NaCl (**A**), 50 or 65 mM CaCl_2_ (**B**). Photographs were taken 3 weeks after germination. Root length of WT and *atcax1*, *atcax4*, and *atcax1/atcax4* plants was measured three weeks after germination. Data represent the mean ± SE of three replicates. Single and double asterisks indicate significant differences from WT at *p* < 0.05 and *p* < 0.01, respectively.

After seed stratification on 1/2 Murashige and Skoog (MS) medium supplemented with different concentrations of NaCl (Figure 3A), assessment of germination indicated that a greater number of wild-type (WT) seeds germinated than seeds of *atcax1*, *atcax4*, and *atcax1/atcax4* double mutants. WT plants also grew better than the transgenic lines on salt-amended media. In contrast, the growth rates of *atcax1*, *atcax4*, *atcax1/atcax4*, and WT did not obviously differ when seeds were germinated and grown on a medium that was not amended with NaCl (Figure 3A). Moreover, root lengths in *atcax1*, *atcax4*, and *atcax1/atcax4* mutants were markedly shorter than those of WT plants when plants were grown in the presence of different concentrations of NaCl (125 and 135 mM) (Figure 3A). The sensitivity of *atcax1*, *atcax4*, and *atcax1/atcax4* mutants to CaCl_2_ was also determined. A 65 mM CaCl_2_ treatment produced an abnormal phenotype in the mutant, while no obvious differences were observed between mutant and WT plants under control conditions (Figure 3B). Overall, the response of *atcax1*, *atcax4*, and *atcax1/atcax4* double mutants to different types of stress indicated that the growth of WT plants was generally better than the growth of *atcax1* and *atcax4* single or *atcax1/atcax4* double mutants. The sensitivity of plants to stress *in vivo* was associated with the deletion of either *AtCAX1* or *AtCAX4* alone or in combination.

**Figure 4:**
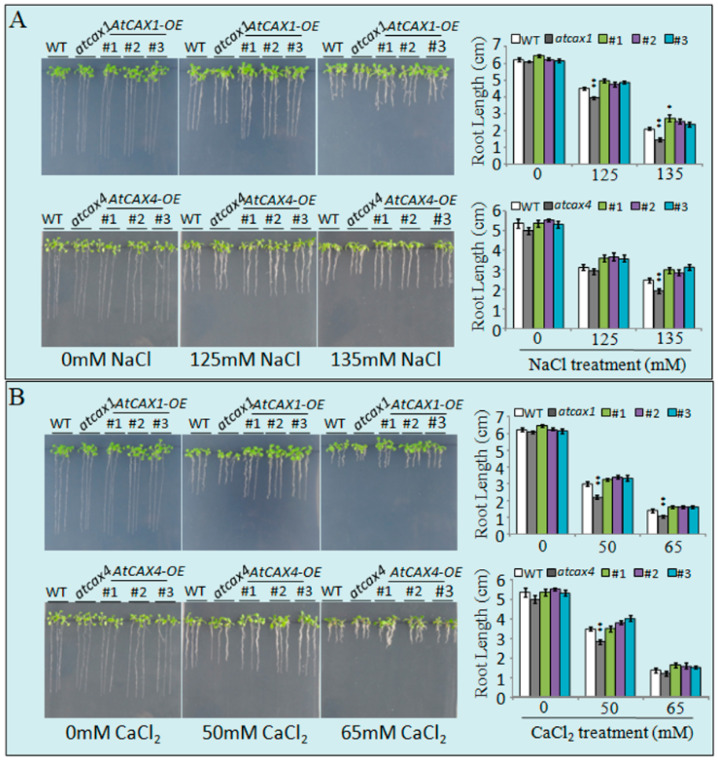
Stress tolerance analysis of *AtCAX1^OE^* and *AtCAX4^OE^* transgenic plants treated with different concentrations of salt and ion stress. Phenotypes of WT, *atcax1*, *atcax4*, and *AtCAX1^OE^* and *AtCAX4^OE^* transgenic seedlings treated with 125 or 135 mM NaCl (**A**), 50 or 65 mM CaCl_2_ (**B**). Photographs were taken 3 weeks after germination. The root length of WT, *atcax1*, *atcax4*, and *AtCAX1^OE^* and *AtCAX4^OE^* transgenic plants were measured 3 weeks after treatment. Data represent the mean ± SE of three replicates. Single and double asterisks indicate significant differences from WT at *p* < 0.05 and *p* < 0.01, respectively.

The response of *AtCAX1* and *AtCAX4* transgenic plants to salt and ion stress was further assessed *in vivo* to determine the effect of the *AtCAX1* and *AtCAX4* interaction on abiotic stress tolerance. Results indicated that the sensitivity of *atcax1* and *atcax4* mutants to NaCl stress increased with NaCl concentration, while the tolerance of plants overexpressing *AtCAX1* or *AtCAX4* to salt stress was significantly enhanced. This was mainly manifested in significantly longer root length in transgenic plants relative to WT plants, while *atcax1* and *atcax4* mutants had the shortest roots (Figure 4A). Similarly, *atcax1* and *atcax4* mutants treated with increasing concentrations of CaCl_2_ exhibited the weakest growth, while *AtCAX1* and *AtCAX4* transgenic plants exhibited better growth than WT plants. At 65 mM CaCl_2_, the growth of all the different lines of plants was inhibited; however, the *AtCAX1* and *AtCAX4* transgenic plants still exhibited better growth than *atcax1* and *atcax4* mutants, as well as WT plants (Figure 4B). Collectively, our results indicate that the overexpression of *AtCAX1* or *AtCAX4* genes can improve abiotic stress tolerance in *Arabidopsis*.

## 3. Discussion

Calcium ions (Ca^2+^) are involved as a second messenger in many physiological processes, where they play a regulatory role in transducing cell signals involved in the growth and stress response of plants. The CAX family is thought to play an important role in regulating intracellular Ca^2+^ and the balance of other cations.

### 3.1. AtCAX1 and AtCAX4 form A Heterodimer

There are six CAX members in *Arabidopsis thaliana*. In the present study, we analyzed the CAX proteins in *Arabidopsis thaliana* for potential interactions using a yeast two-hybrid system. Results demonstrated that CAX1 and CAX4 proteins interact, which was verified by the co-transformation of yeast strains and BiFC assays (Figure 1). Previous studies revealed that an *atcax1/atcax3* double mutant exhibited slower growth (shorter plants), relative to WT and single mutants, when planted in soil. When seeds were germinated and grown on a culture medium amended with the different levels of Ca^2+^, the *atcax1/atcax3* double mutant exhibited a higher sensitivity to Ca^2+^, exhibiting poorer growth, producing shorter plants, and a greater level of leaf tip necrosis, relative to WT and single mutant plants. Notably, the growth rate of the *atcax1/atcax3* double mutant on a medium containing 15 mM MgCl_2_ was better than the control and the *atcax3* single mutant, while the poor growth rate of the *atcax1/atcax3* double mutation was improved by MgCl_2_ [24]. In addition, the expression level of *AtCAX3* and *AtCAX4* in *Arabidopsis thaliana* was significantly increased in *atcax1* inserted mutant plants [22]. These studies indicate that a potential interaction occurs between CAX members. The N-terminal regulatory region (NRR) of CAXs function as an autoinhibitory region in yeast and possess an autoinhibitory domain for Ca^2+^/H^+^ transport activity. The interaction of AtCAX1 (N terminal, AtCAX1-N) and AtCAX4 (N terminal, AtCAX4-N) was detected in the Y2H system used in the current study. The Ca^2+^ transport capacity of full-length AtCAX1 can be specifically activated by the CAX-interacting protein, CXIP4 [33]. The C-terminal region also has a regulatory function in some transporters, such as the mammalian Na^+^/H^+^ exchanger isoform 1 (NHE1) and the cyanobacterial Na^+^/H^+^ antiporter [34,35,36]. Our results indicate that AtCAX1 interacts with the N-terminal region of AtCAX4 and provides evidence that CAXs are capable of forming a heterodimeric protein through an interaction at the N-terminal region.

### 3.2. AtCAX1 and AtCAX4 Interact in Plants in Response to Environmental Stress

We investigated the expression pattern of *AtCAX1* and *AtCAX4* in *Arabidopsis* in response to different salt and ion treatments. In addition to the effect of NaCl, we also assessed the effect of Ca^2+^ treatments on the expression of *CAX1* and *CAX4*. Results indicated that *CAX1* and *CAX4* expression was induced to different degrees in roots and leaves (Figure 2). This finding is consistent with a previous study reporting that *CAX4* mRNA levels increased in response to a salt treatment [26]. CAX1 in *Arabidopsis* is highly expressed in leaf tissue and modestly expressed in roots, stems, and flowers [24]. *CAX4* expression is relatively low in most tissues, relative to *CAX1*; however, when expressed at high levels in plants, its biochemical properties resemble other CAXs [17,26]. Previous studies, whose main focus was the response of CAX expression to Ca^2+^, have demonstrated that CAX1 and CAX4 are the main Ca^2+^ transporters in plants. However, in the present study, the response to NaCl was also analyzed, in addition to Ca^2+^. Our analysis of stress-induced expression indicated that CAX1 and CAX4 are involved in abiotic stress response in general, including the response to salt and ion stress (Figure 2). Bioinformatic analysis of *Arabidopsis* CAX family members revealed that AtCAX1 and AtCAX4 belong to the CAX IA developmental group, suggesting that the target of these two proteins may be similar or the same; CAX4 is most closely related to CAX1 at the amino acid level [11,25]. The loss-of-function mutant (*cax4-1*) and *CAX4* RNA interference (*CAX4* RNAi) lines were reported to exhibit altered root growth and development in response to Cd^2+^, Mn^2+^, and auxin [17]. These results suggest that CAX4 functions in root growth in plants under heavy metal stress conditions. Our current study also confirms that the *CAX4* is most highly expressed in roots (Figure 2). Although *CAX1* is most highly expressed in leaves, both *CAX1* and *CAX4* were induced to varying degrees in both roots and leaves in response to ion stress conditions (Figure 2). The growth of *cax1*, *cax4,* and *cax1/cax4* mutants treated with stress levels of salt and ion was analyzed with a specific focus on root elongation. Results indicated no significant differences in root and shoot growth between *cax1*, *cax4,* and *cax1/cax4* mutants under normal conditions, although the growth of wild-type plants was better than the mutant plants when plants were exposed to different cations (Figure 3). Previous studies demonstrated that CAXs can play compensatory roles in many cell functions. For example, plants that have lost a single CAX transport function are almost indistinguishable from the wild-type plants under normal growth conditions [11], but plants display severe growth defects in some double CAX deletion mutants, implying the compensatory function of CAX gene members [18,23]. These observations also imply that the interaction between CAX members is critical for plant growth, especially under abiotic stress conditions.

In the present study, we provide the first data demonstrating the response of *cax1*/*cax4* to Ca^2+^. Notably, the overexpression of *CAX1* or *CAX4* genes can significantly improve plant tolerance to abiotic stress, relative to *cax1* and *cax4* mutants (Figure 4), indicating that overexpression may enhance the interaction between CAX1 and CAX4 in vivo, which leads to enhanced abiotic stress tolerance, and the interaction between CAX1 and CAX4 may be crucial to the enhancement of the tolerance of plants to abiotic stress. Changes in the relative expression of *CAX* transcripts in response to abiotic stresses have been previously reported. Studies in *Arabidopsis* and rice have indicated that *CAX* transcripts can either increase or decrease in response to dehydrative stresses, including drought, heat, cold, and salinity [37,38]. Enhanced *CAX* expression in plants in response to salt stress suggests that these transporters play a functional role in the response to salt stress [7,39]. CAX proteins are involved in a variety of abiotic stress response pathways, including as a modulator of cytosolic Ca^2+^ signaling [39]. The interaction between CAX1 and CAX4 may alleviate the self-inhibition resulting form the N-terminal region of CAX proteins. Thus, the role of the heterodimer in the regulation of intracellular Ca^2+^ signaling in plants is worthy of further study.

## 4. Materials and Methods

### 4.1. Yeast Two-Hybrid (Y2H) Screening

Y2H experiments were performed with the Matchmaker™Gold Yeast Two-Hybrid System as described by the manufacturer (Clontech Laboratories, CA, USA). Full-length *AtCAX1* and *AtCAX4*, as well as the N-terminal truncated 108 aa of *AtCAX1* (∆*NAtCAX1* or *AtCAX1-C*) or *AtCAX4* (∆*NAtCAX4* or *AtCAX4-C*) and C-terminal truncated 36 aa sequences of *AtCAX1* (∆*CAtCAX1* or *AtCAX1-N*) or *AtCAX4* (∆*CAtCAX4* or *AtCAX4-N*) were amplified using the primers listed in Table S1 and cloned into the pGBKT7 and pGADT7 vectors. The constructs, pGBKT7-AtCAX1 and pGADT7-AtCAX4, were co-transformed into the *Saccharomyces cerevisiae* strain in the Y2HGold and cultured on SD media lacking leucine and tryptophan (SD/-Leu/-Trp) and SD media lacking leucine, tryptophan, histidine, and adenine and containing Aureobasidin A (AbA) (SD/–Leu/–Trp/–His/–Ade+AbA), as described in [40]. The interacting CAX1 and CAX4 proteins were detected on the SD/–Leu/–Trp/–His/–Ade+AbA+X-a-Gal medium to assess the expression of four reporter genes (*HIS3*, *ADE2*, *AUR1-C*, and *MEL1*), which were activated by a positive interaction between interacting CAX proteins.

### 4.2. Bimolecular Fluorescence Complementation (BiFC) Assay

The vector for BiFC was constructed by replacing the GFP in pBS-35S-GFP with the N-terminus (154 aa) or C-terminus (80 aa) of the mVenus plasmid, yielding pBS-35S: VN154 (nGFP) and pBS-35S:VC80 (cGFP), respectively [41]. Full-length *AtCAX1* and *AtCAX4*, as well as the N-terminal truncated of *AtCAX1* (∆*NAtCAX1* or *AtCAX1-C*), and C-terminal truncated sequences of *AtCAX1* (∆*CAtCAX1* or *AtCAX1-N*) or *AtCAX4* (∆*CAtCAX4* or *AtCAX4-N*) were amplified using the primers listed in Table S1 and cloned into the pBS-35S: VN154 (nGFP) and pBS-35S:VC80 (cGFP) vectors, generating pBS-35S: AtCAX1-VC80 (AtCAX1-cGFP) and pBS-35S: AtCAX4-VN154 (AtCAX4-nGFP), pBS-35S: AtCAX1-VC80-N (AtCAX1-N-cGFP), pBS-35S: AtCAX1-VC80-C (AtCAX1-C-cGFP), and pBS-35S: AtCAX4-VN154 (AtCAX4-N-nGFP). A mixture of an nGFP construct and a cGFP construct (500 ng each) (AtCAX4-nGFP+AtCAX1-cGFP, AtCAX4-N-nGFP+ AtCAX1-N-cGFP, AtCAX4-N-nGFP+AtCAX1-C-cGFP) was used for particle bombardment to co-express proteins of interest in onion epidermal cells [42]. Images were viewed and recorded on a confocal scanning-laser imaging system (Olympus Fluoview, FV500).

### 4.3. Plant Material and Transformation

*Arabidopsis thaliana* ecotype Columbia-0 (Col-0) was used in this study as wild-type (WT), T-DNA insertion mutants, and to create transgenic plants. The *AtCAX1* and *AtCAX4* T-DNA insertion mutant SALK-108310 (*atcax1*) and SALK-201217C (*atcax4*) were obtained from the Arabidopsis Biological Resource Center (ABRC: http://www.arabidopsis.org/). The mutant lines *atcax1* and *atcax4* were crossed to generate double mutants (*atcax1/atcax4*), and homozygous double mutant plants were screened from the resulting F2 progeny by PCR. Homozygous mutant plants were identified using the gene-specific primers and T-DNA left border-specific primers listed in Table S1.

The following *Arabidopsis* (*Arabidopsis thaliana*) transgenic plants were generated: 35S::AtCAX1/Col-0 (AtCAX1^OE^), and 35S::AtCAX1/Col-0 (AtCAX4^OE^). The full-length coding sequence of *AtCAX1* and *AtCAX4* were separately amplified and individually ligated into the pBI121 vector under the control of the 35S promoter to generate transgenic *AtCAX1^OE^* and *AtCAX4^OE^* constructs. The resulting plasmids were introduced into wild-type *Arabidopsis thaliana*, ecotype Columbia plants using a floral dip infiltration method for *Agrobacterium tumefaciens*-mediated transformation [43]. Homozygous lines were identified and used in the subsequent analyses. The primers used for the generation of the constructs are listed in Table S1. Plants were grown under a 16-h light/8-h dark cycle at 22 °C.

For the stress tolerance assay, 30 seeds of Col-0, *atcax1*, *atcax4, atcax1/atcax4* mutants, *AtCAX1^OE^,* and *AtCAX4^OE^* were surface sterilized and placed on 1/2 Murashige and Skoog (MS) medium supplemented with different concentrations of NaCl (125 and 135 mM), or CaCl_2_ (50 and 65 mM). After 14 days, seedling phenotypes were photographed, and the root length of the seedlings were measured. The experiment was repeated three times. Significant statistical differences between treatment means were determined at *p* < 0.05 using a Student’s *t*-tests.

### 4.4. RNA Isolation and Northern Blotting

Total RNA was isolated using a RNeasy plant Mini kit (Qiagen, Hilden, Germany) and treated with RNasefree DNaseI (Qiagen, Hilden, Germany). First-strand cDNA was synthesized using SuperScript III reverse transcriptase (Invitrogen, Carlsbad, CA, USA). Pairs of gene-specific primer pairs, *AtCAX1*-RT-FW and *AtCAX1*-RT-RV were used for *AtCAX1*, *AtCAX4*-RT-FW and *AtCAX4*-RT-RV were used for *AtCAX4*, while *Actin*-FW and *Actin*-RV were used to amplify the Actin gene (Table S1). Transcript abundance was determined by RT-qPCR using SYBR green I on a LightCycler^®^480 system II (Agilent Technologies, Palo Alto, CA, USA).

For northern blotting analysis, total RNA (10 µg) obtained from transgenic *Arabidopsis* lines was separated on a 1% (*m/v*) agarose–formaldehyde gels and transferred to Hybond N^+^ membranes. Hybridizations were carried out at 50 °C using *AtCAX1* and *AtCAX4* probes labeled with digoxigenin (DIG, Roche, Basel, Switzerland). Signals were detected with CDP-Star using a luminescent image analyzer (Fujifilm, LAS-4000mini, Tokyo, Japan). The single lines were named #1, #2, and #3, respectively.

### 4.5. Analysis of Gene Expression Using Reverse Transcription–Quantitative PCR (RT-qPCR)

Gene expression was quantified by RT-qPCR. *Arabidopsis* seeds were surface sterilized and placed on solid half MS medium. After 2 days of stratification at 4 °C, the plates were moved to a 22 °C incubator for propagation. The seedlings were transferred from the plates to a 1:1 mixture of soil and vermiculite and grown to maturity at 22 °C. The plants were grown under a 16-hlight/8-h-dark cycle in a growth chamber. Roots, stems, leaves, panicle, and siliques of two-month-old plants were sampled and used in the RT-qPCR analysis. A second batch of seedlings was pre-cultured for 2 weeks on 1/2 solid medium and then treated with different concentrations of NaCl (125 and 135 mM) and CaCl_2_ (50 and 65 mM). Shoots and roots were sampled after 0, 6, 12, and 24 h after treatment and used in the RT-qPCR analyses.

## 5. Conclusions

In the present study, the interaction between the N-terminal region of *AtCAX4* with *AtCAX1,* CAX family members in *Arabidopsis,* was demonstrated using a yeast two-hybrid system and a bimolecular fluorescence complementation assay. This is the first study to report the interaction between CAX1 and CAX4 in plants. Further experiments demonstrated that the *AtCAX1* and *AtCAX4* expression is induced in roots and leaves in response to abiotic stress (NaCl and Ca^2+^). Functional analysis of *AtCAX1* and *AtCAX4* in *Arabidopsis* demonstrated that *atcax1*, *atcax4* single mutants, and *atcax1/atcax4* double mutants were more sensitive to abiotic stress than WT plants. In contrast, the overexpression of *AtCAX1* or *AtCAX4* in *Arabidopsis* improves abiotic stress tolerance. AtCAX1 and AtCAX4 may form a heterodimer in plants in response to abiotic stress.

## Data Availability

Not applicable.

## Supplementary Materials

Supplementary Materials can be found at https://www.mdpi.com/1422-0067/22/2/856/s1. Table S1: Sequence of the primers used for PCR. Figure S1. C-terminus sequence of pGBKT7-AtCAX1 and C-terminus sequence of pGADT7-AtCAX4 were co-transformed into yeast Y2HGold cells. Transformed yeast cells were grown on SD-Leu-Trp and SD-Leu-Trp-His-Ade+X-a-gal+AbA medium for 2–3 days at 30 °C. Figure S2. Characterization of *atcax1* and *atcax4* T-DNA insertion mutants, and *atcax1*/*atcax4* homozygous double mutants. (A) T-DNA insertion site in *AtCAX1* and *AtCAX4*; gray boxes represent exons; black lines represent introns; (B) Reverse-transcription-quantitative PCR (RT-qPCR) analysis confirmed the knockout status of *atcax1*, *atcax4*, and homozygous double mutants *atcax1*/*atcax4*; Actin expression was used as an internal control in the RT-qPCR analysis; (C) RNA gel blot analysis of T3 transgenic plants expressing *AtCAX1* or *AtCAX4*. WT: *Arabidopsis thaliana* ecotype Columbia-0; #1, #2, and #3: T3 seedlings expressing *AtCAX1* or *AtCAX4* in a Columbia-0 background.

## Abbreviations

CAXs	Ca^2+^/H^+^ antiporters
BiFC	Bimolecular fluorescence complementation
Ca^2+^	Calcium ion
Ba^2+^	Barium ion
AbA	Aureobasidin A
MS	Murashige and Skoog
