# Supplementary material for: Description of AtCAX4 in Response to Abiotic Stress in Arabidopsis
Source: Int J Mol Sci. 2021 Jan 16;22(2):856. doi: 10.3390/ijms22020856 (PMC7830611; doi:10.3390/ijms22020856)
Supplement: Supplementary file 1 [file ijms-22-00856-s001.pdf]

International Journal of
Molecular Sciences

MDPI

Table S1. Sequence of the primers used for PCR

Primers	Sequence (5'-3')
pGBKT7-AtCAX1-F	GAATTCATGGCGGGAATCGTGACAGAG (*Eco*RI)
pGBKT7-AtCAX1-R	GGATCCTTAACCCGTTTTAACTTTATTT (*Bam*HI)
pGADT7-AtCAX4-F	GAATTCATGTCTTCAATCAGTACGGAAT (*Eco*RI)
pGADT7-AtCAX4-R	GGATCCTTACCTTTTCGTTATTGTATG (*Bam*HI)
nYFP-AtCAX1-F	GGTACCAGTAGAGAAATGGCGGGAAT (*Kpn*I)
nYFP-AtCAX1-R	ACTAGTACCCGTTTTAACTTTATTTG (*Spe*I)
cYFP-AtCAX4-F	GTCGACAGATGTCTTCAATCAGTACG (*Sal*I)
cYFP-AtCAX4-R	ACTAGTCCTTTTCGTTATTGTATGAT (*Spe*I)
nYFP-AtCAX1-N-F	GGTACCATGGTGGGATACTTGAAAAAC (*Kpn*I)
nYFP-AtCAX1-N-R	ACTAGTGACATTGTTCATCGCTTGATGTC (*Spe*I)
nYFP-AtCAX1-C-F	GGTACCATGTCTTCTTCTTCTTTGAGG (*Kpn*I)
nYFP-AtCAX1-C-R	ACTAGTACCCGTTTTAACTTTATTTG (*Spe*I)
cYFP-AtCAX4-N-F	GTCGACATGTCTTCAATCAGTACGGA (*Sal*I)
cYFP-AtCAX4-N-R	ACTAGTTTCGGTTGAGGGTGAGTTAG (*Spe*I)
pBI121-AtCAX1-F	GGATCCTCAGTAGAGAAATGGCGGGA (*Bam*HI)
pBI121-AtCAX1-R	GAGCTCTCCATTGTCTCTCGCTTTGG (*Sac*I)
pBI121-AtCAX4-F	GGATCCAAGATGTCTTCAATCAGTAC (*Bam*HI)
pBI121-AtCAX4-R	GAGCTCTCGAGTTACCTTTTCGTTAT (*Sac*I)
AtCAX1-RT-F	TCAGTAGAGAAATGGCGGGA
AtCAX1-RT-R	TCCATTGTCTCTCGCTTTGG
AtCAX4-RT-F	GTCGACAGATGTCTTCAATCAGTACG
AtCAX4-RT-R	ACTAGTCAACACAACTACATCTGACC
atcax1-LP	ATGCGATAACGAGTGTCATCC
atcax1-RP	ATGGACCAATATTCTTTGGGG
atcax4-LP	TGGAGGCTGAAGAATACGATG
atcax4-RP	TGCCACTAACACTGTCACTGG
LBb1.3	ATTTTGCCGATTTCGGAAC

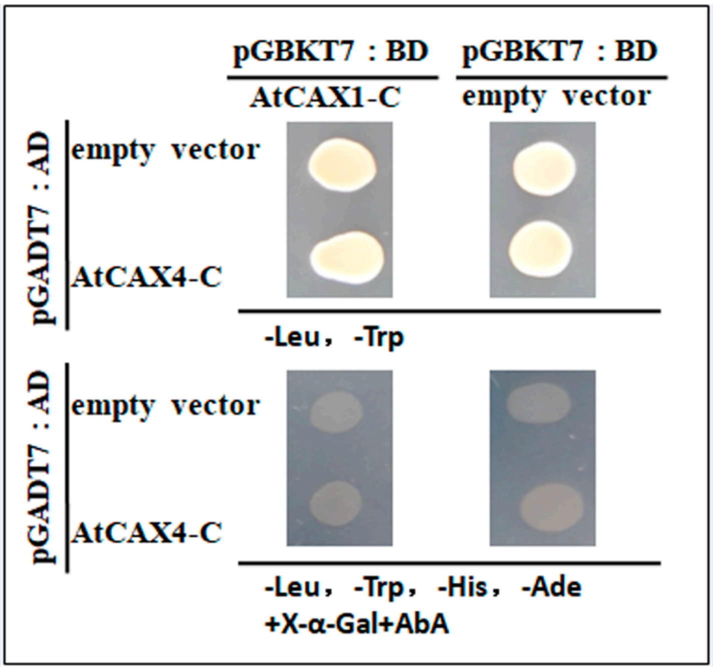

Figure S1. C-terminus sequence of pGBKT7-AtCAX1 and C-terminus sequence of pGADT7-AtCAX4 were co-transformed into yeast Y2HGold cells. Transformed yeast cells were grown on SD-Leu-Trp and SD-Leu-Trp-His-Ade+X-a-gal+AbA medium for 2–3 days at 30°C.

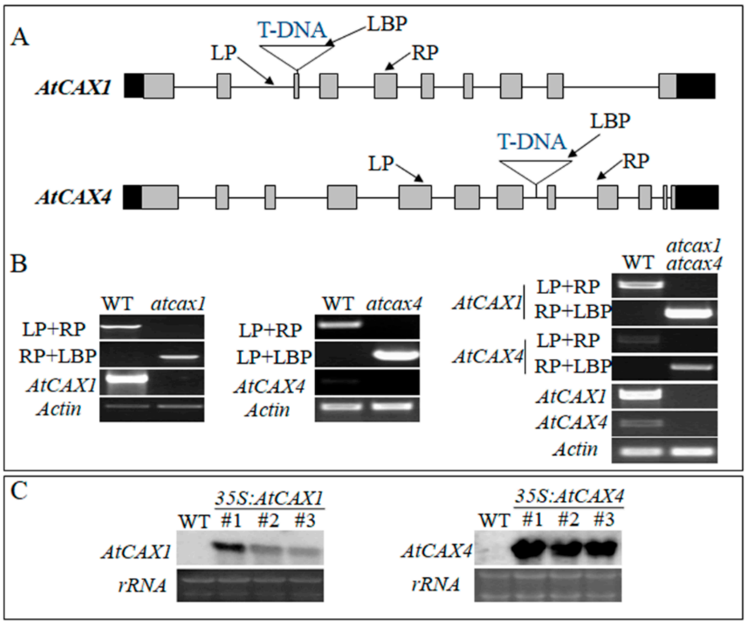

Figure S2. Characterization of *atcax1* and *atcax4* T-DNA insertion mutants, and *atcax1*/*atcax4* homozygous double mutants. (A) T-DNA insertion site in *AtCAX1* and *AtCAX4*; gray boxes represent exons; black lines represent introns; (B) Reverse-transcription-quantitative PCR (RT-qPCR) analysis confirmed the knockout status of *atcax1*, *atcax4*, and homozygous double mutants *atcax1*/*atcax4*; Actin expression was used as an internal control in the RT-qPCR analysis; (C) RNA gel blot analysis of T3 transgenic plants expressing *AtCAX1* or *AtCAX4*. WT: *Arabidopsis thaliana* ecotype Columbia-0; #1, #2, and #3: T3 seedlings expressing *AtCAX1* or *AtCAX4* in a Columbia-0 background.
